# Successfully Anticipated Difficult Airway Management of a “Can Ventilate, but Cannot Intubate” Situation for Urgent Laparoscopic Appendectomy in a Patient with Duchenne Muscular Dystrophy

**DOI:** 10.3390/reports7020047

**Published:** 2024-06-14

**Authors:** Fabian P. Brunner, Philippe Neth, Alexander Kaserer

**Affiliations:** Institute of Anesthesiology, University and University Hospital Zurich, Raemistrasse 100, 8091 Zurich, Switzerland

**Keywords:** difficult airway, Duchenne, difficult intubation, reversal of anesthesia, advanced airway management, wake-up, Aintree

## Abstract

Background: Airway management in Duchenne patients can be challenging. We present a case of an anticipated difficult airway in a 24-year-old Duchenne patient that was managed by planning different suitable strategies based on the unanticipated difficult airway algorithm of the Difficult Airway Society (DAS). Case presentation: The patient initially presented with appendicitis, requiring a laparoscopic appendectomy within 6 h. Due to the underlying condition and a known difficult airway, we anticipated potential airway problems and successfully managed the “can ventilate but cannot intubate” situation using the algorithm. The difficult airway was attributed to reduced mandibular mobility, limited inclination or reclination, a large tongue, prominent incisors, and a posteriorly positioned epiglottis. Despite thorough preparation and team briefing, we experienced three failed intubation attempts. Considering limited nighttime resources, the urgency of the surgery, the need for a tube for laparoscopy, and the risk of exacerbating airway issues, we made the decision to awaken the patient and wait for a second attempt after the epiglottis swelling had subsided. We used reversible, short-acting agents for induction, enabling us to continue with the algorithm within the allotted timeframe. In a second stage, we successfully performed fiberoptic-guided intubation via a supraglottic airway device using the Aintree intubation catheter, utilizing more favorable resources. Conclusions: For a patient with Duchenne muscular dystrophy and a difficult airway, advanced expertise is critical. Detailed anesthesia planning, clear team communication, and the use of reversible, short-acting agents are crucial. Adherence to the Difficult Airway Society guidelines is essential for safe airway management.

## 1. Background

Anesthesiologic management in Duchenne patients is complex for potential airway problems and several other reasons. Duchenne muscular dystrophy (DMD) is caused by a faulty dystrophin protein, which is approximately in 19.8 out of 100,000 male births worldwide and is wrongly encoded by the DMD gene on chromosome X [1]. The following inability to maintain the sarcolemma of muscle cells presents typically with progressive muscular atrophy. Therefore, not only the avoidance of suxamethonium [2] and halogenated inhalational anesthetics [3,4,5], the risk of sudden cardiac arrest [3,4,5] in undiagnosed patients or the decreased ability to cough in the perioperative period play a role [6], but also the airway management in Duchenne patients can be challenging due to the muscular contractions [3,4,7]. This case highlights the need to assess a potentially difficult airway during pre-anesthesia assessment, particularly in patients with muscular dystrophy, and therefore the careful preparation and evaluation of the difficult airway algorithm in the context of the patient’s medical needs in addition to human and clinical resources [8]. The DAS-difficult airway algorithm [9] gives clear guidance for an unanticipated difficult airway but was used in this context to anticipate and manage the induction of a pre-assessed potentially difficult airway in a Duchenne patient. The described exit strategy of “waking up” the patient [9] is an option that must be considered in a “can ventilate but cannot intubate” situation if set in context to available resources and the patient’s condition and need for medical treatment. This option enables us to continue the algorithm within the given time frame by clinical indication in a setting with more favorable resources [8]. To enable a potential exit strategy, reversible agents such as rocuronium [10] and potent ultra-short-acting selective μ-opioid agonists such as remifentanil [11] were used.

## 2. Case Presentation

We present a challenging case involving a 24-year-old male Duchenne patient (BMI of 18.7 kg/m^2^) with an American Society of Anesthesiologists (ASA) score of 4, who arrived at our Emergency Department (ED) with lower right abdominal pain that had begun 24 h earlier. The patient had no fever but exhibited elevated infection parameters indicative of acute appendicitis, a diagnosis confirmed by a CT scan that showed no signs of perforation. This patient’s condition was complicated by the deletion of exon 12 in the dystrophin gene, leading to all his complicating clinical aspects, such as cognitive impairment, cardiomyopathy, contractions in all flexor muscles, scoliosis, and progressive weakening of the respiratory muscles. Consequently, the patient had previously undergone thoracic spondylodesis and required continuous BIPAP (room air, PEEP 4 mbar, Psupp 14 mbar) with their own device. It was evident that he would need respiratory support in the ICU post-surgery. By the time the decision was made that surgery was necessary within 6 h, the limited night teams were already occupied with other surgical activities of higher priority.

Airway assessment according to current guidelines [5,6] revealed several concerning findings: reduced mouth opening (1–2 cm) indicating limited mandibular mobility, inability to incline or recline, Mallampati Class 3, a large tongue, prominent upper front teeth (teeth 7–10), and the absence of nasopharyngeal imaging examination. The patient provided a handwritten anesthesia protocol from six years earlier, reporting a Cormack & Lehane (CL) Grade 3 with failed direct laryngoscopy but successful fiberoptic oral intubation and adequate mask ventilation.

The decision to proceed with surgery involved interdisciplinary discussions, considering various options such as awake fiberoptic guided intubation while also taking into account the patient’s emotional distress and respiratory challenges. Based on our archive records of our patient, successful mask ventilation and asleep fiberoptic intubation were achievable, but video laryngoscopy, especially with a D-Blade (hyper angulated blade), had not been attempted yet. Maintenance of oxygenation and avoiding aspiration was our highest priority throughout our care, which is why two suctions and a set for front-of-neck access were always available.

We decided to adhere to the DAS difficult airway algorithm, initiating a modified rapid sequence induction (RSI) as Plan A, with the use of potentially reversible agents and video laryngoscopy employing a MAC #4 blade. The choice for a modified RSI was made to confirm the effectiveness of mask ventilation before proceeding further, considering the patient’s Duchenne dystrophy, respiratory status, and the need for swift intubation. 

Plan B involved switching to the D-Blade with the potential use of a bougie. Adapting the DAS algorithm to our needs, Plan C consisted of a combination of laryngoscopy to lift the tongue and fiberoptic-guided oral intubation. 

Given the high success rate and routine use of video laryngoscopy, we regarded it as a more favorable option than fiberoptic intubation, designating it as Plan C. 

In the event that ventilation remained possible, “waking up” the patient was determined to be Plan D. 

Several considerations influenced this decision, including limited human resources during the night shift, the urgency of the surgery, the need for a tube for laparoscopy, the absence of ENT specialists at that hour, and the likelihood of further airway complications with additional manipulations, such as fiberoptic-guided intubation through a supraglottic airway device using an Aintree intubation catheter [10]. This scenario constituted Plan E, which we wished to reserve as the last resort before resorting to a more suitable setting with greater manpower, broader expertise, a refreshed team, and a patient who had time to recover from Plans A to D, provided that the surgical urgency allowed for this timeframe.

For induction, we administered reversible agents at state-of-the-art RSI doses. Fentanyl served as the primary opioid, supplemented with fast-acting remifentanil. Propofol, following Schnider’s TCI model, served as the hypnotic agent of choice. To adhere to Duchenne contraindications, we avoided succinylcholine and selected Rocuronium as the muscle relaxant, with ready-to-use, drawn-up Sugammadex for reversal. In anticipation of hemodynamic depression, particularly given Duchenne-related cardiomyopathy, we prepared a noradrenaline perfusor in advance. 

Preoxygenation was performed using the patient’s BIPAP device, set to 100% FiO_2_. We initiated a modified RSI, minimally supported by noradrenaline for hemodynamic stability, which we continuously monitored via an arterial catheter. Neuromuscular blockade was confirmed through relaxometry. Despite challenges in head positioning due to muscular dystrophy, we successfully achieved two-handed mask ventilation. However, Plan A failed as video laryngoscopy revealed a Cormack–Lehane grade 3b view with no glottic visibility, a posteriorly positioned larynx, hindered by prominent incisors, and limited mouth opening. As the same view could be obtained at direct laryngoscopy six years ago, disease progression regarding the airway is postulated. Recognizing that we were in a “can ventilate but cannot intubate” situation, we requested ENT assistance and prepared for Plan B while maintaining oxygenation above 90% to prevent desaturation. Utilizing a D-Blade, we obtained a partial view of the arytenoid cartilage, but the vocal cords remained obscured. During the laryngoscopy maintaining this view, a direct intubation attempt to place the tube as well as one using a bougie were unsuccessful. 

Proceeding to Plan C, we once again utilized the D-Blade to create space for fiberoptic guided intubation. Unfortunately, swelling obscured the epiglottis, leading the scope into the esophagus. ENT confirmed epiglottal swelling the following day. We decided to awaken the patient to avoid front-of-neck access, considering surgery urgency, airway risks, and limited resources overnight. During this process, the patient maintained stable hemodynamics without experiencing aspiration, desaturation, or hypercapnia. 

We discontinued the administration of Propofol and Remifentanil, reversed the effects of Rocuronium using Sugammadex, and administered Nalbuphine to counteract the effects of Fentanyl. Additionally, Methylprednisolone was administered to prevent further swelling. The patient awakened without any incidents and was subsequently transferred to the intensive care unit with BIPAP support. This delay allowed for additional evaluations, including an airway CT scan and an assessment by ENT specialists, which confirmed the previously identified anatomical challenges. Plans were put in place for a subsequent attempt with Plan E, to be carried out by an ENT anesthesia team during the morning shift.

The second induction used the same agents and dosages to facilitate the placement of a size #3 supraglottic device, one size smaller than ideal due to the patient’s limited mouth opening. Although ventilation was only possible with a significant leak, fiberoptic-guided intubation through the device was performed using an Aintree intubation catheter. The Aintree’s correct placement was verified via a fiberoptic view of the carina. After removing the supraglottic device while maintaining the Aintree’s position, the tube was inserted over it into the trachea, as confirmed by capnography and bronchoscopy. The laparoscopic appendectomy was initially attempted but converted to a laparotomy owing to the patient’s challenging anatomy.

Post-operatively, the patient was assessed by ENT, anesthesia, and intensive care teams due to increased glottic swelling. Extubation, conducted 48 h after intubation, revealed left-sided recurrent nerve palsy, which resolved in the following weeks. Within the first three days at the hospital, he developed pneumonia caused by Klebsiella, worsening his respiratory condition. This led to emergency re-intubation 48 h post-extubation, where the same anesthesia team successfully implemented Plan E for airway management. A dilatative tracheotomy was performed 13 days into the hospital stay as he persistently needed CPAP while being at high risk for aspiration. After a 30-day hospitalization, the patient was discharged for rehabilitation and to learn tracheostoma management.

## 3. Discussion

Several key points emerge from this case. First, preoperative airway evaluation, both clinical and from prior anesthetic experiences, is crucial for anticipating and managing potential risks [12,13]. Adhering to established guidelines [9,14], maintaining clear communication [8], and being flexible in decision-making are essential for effectively handling such situations [15]. Ideally, the team knows each other well and is trained to work together.

The selection of a modified Rapid Sequence Induction (RSI) approach in the context of a potentially challenging airway [16], utilizing short-acting Remifentanil [11] and Rocuronium, with the option of reversal using Sugammadex [10], aligns with established practices documented in the literature. Notably, Succinylcholine is contraindicated in individuals with Duchenne muscular dystrophy [2] due to the associated risks.

Additionally, it’s worth mentioning that Nalbuphine is a well-established agent for counteracting ventilatory depression induced by fentanyl [17], further underscoring the importance of judicious drug selection and the need for tailored pharmacological strategies in patients with Duchenne muscular dystrophy and potential airway challenges [3,4,5,7].

A point of contention revolves around the decision to opt for video laryngoscopy, despite previous difficulties encountered with direct laryngoscopy in this patient, as opposed to initially considering fiberoptic intubation during sleep. However, recent studies provide compelling evidence supporting the effectiveness of video laryngoscopy in similar scenarios [18,19], especially when augmented with a D-Blade [20] and a bougie [21]. It is evident that routine use of video laryngoscopy, better visibility of the airway for all team members, and less airway trauma will be beneficial in this case [18], This approach finds further validation in a case report detailing the successful use of video laryngoscopy and a bougie in a Duchenne muscular dystrophy patient [3]. 

Conversely, fiberoptic nasal intubation was not attempted due to the associated risks of hemodynamic changes and prolonged intubation times [22], compounded by the risk of bleeding in the context of the patient’s challenging anatomy. The decision to reserve oral fiberoptic intubation as our Plan C can be open to discussion, considering that the manipulation involved induced swelling, making the attempt more challenging. Notably, studies comparing video laryngoscopy to the gold standard in awake intubations have demonstrated comparable or even faster intubation times and success rates [23], lending support to our decision to employ video laryngoscopy in managing a sleeping patient with a difficult airway. Furthermore, we saw a potential problem in an awake fiberoptic intubation in terms of our patient’s compliance, resulting potentially in aspiration or laryngospasm. 

Interdisciplinary discussion is essential to assess the appropriateness of the chosen exit strategy and to consider whether alternative actions, such as implementing Plan E during the initial induction, should have been contemplated earlier. The clinical presentation of the epiglottis being situated entirely on the posterior side of the pharynx led us to believe that using a supraglottic device might exacerbate trauma to the epiglottis by pushing it downward, potentially obstructing the glottis completely. Consequently, we regarded front-of-neck access as the last resort.

The initial unsuccessful intubation attempts likely played a role in the development of recurrent nerve palsy, a complication consistent with the literature documenting glottic edema as a common occurrence [24]. Additionally, the broader challenges associated with airway management in patients with muscular dystrophy, such as the heightened risks of anesthesiologic complications like rhabdomyolysis and potentially life-threatening hyperkalemia when using succinylcholine, have been well documented [3,5].

## 4. Conclusions

In conclusion, managing a patient with Duchenne muscular dystrophy who presents with a challenging airway requires the expertise of senior medical professionals. Thorough anesthetic planning and effective interdisciplinary communication are essential prerequisites before proceeding with intubation. The use of reversible and short-acting agents is crucial for maintaining flexibility in the exit strategy. Furthermore, the DAS (Difficult Airway Society) algorithm, designed for unanticipated difficult airways, not only proves invaluable for responding to unexpected airway challenges but also serves as a valuable tool for anticipating and preparing for difficult airway scenarios in such patients.

## Data Availability

The clinical records analyzed during this case report are not publicly available due to a duty of confidentiality but are available from the corresponding author on reasonable request. Our clinical record was collected in the clinical information system of the University Hospital Zurich.
